# The impact of different anticoagulants and antiplatelets regimens on acute epistaxis outcomes

**DOI:** 10.1007/s00405-024-08718-6

**Published:** 2024-05-23

**Authors:** Elchanan Zloczower, Sapir Pinhas, Raviv Allon, Adi Syn-Hershko, Tom Raz Yarkoni, Maayan Marom, David Kiderman, Oded Cohen, Meir Warman

**Affiliations:** 1 Department of Otolaryngology, Head and Neck Surgery, Kaplan Medical Center, Rehovot, Israel; 2 Faculty of Medicine, The Hebrew University of Jerusalem, Jerusalem, Israel; 3 Joyce and Irvin Goldman Medical School, Faculty of Health Sciences, Ben Gurion University of the Negev, Beer-Sheba, Israel; 4 Division of Otorhinolaryngology, Faculty of Health Sciences, Soroka University Medical Center, Ben-Gurion University of the Negev, Beer-Sheba, Israel

**Keywords:** Acute epistaxis, Anticoagulation, Antiplatelet, New oral anticoagulation

## Abstract

**Background:**

The impact of anticoagulants (AC) and antiplatelets (AP) on the management of acute epistaxis remains unclear. This study investigated the association between AC/AP therapy and treatment outcomes in patients with acute epistaxis.

**Methodology:**

A retrospective analysis of patients presented to the otolaryngology emergency room with acute epistaxis (2014–2022). Patients were categorized based on their regular medications: AP, dual AP therapy (DAPT), new oral anticoagulants (NOAC), vitamin K antagonists (VKA), or no regular AC/AP use (control group). Outcome measures included rates of minor interventions (chemical or electrical cautery, nasal tamponade), major interventions (endoscopic ligation, embolization), recurrent emergency department visits, admission rates, and duration.

**Results:**

786 patients were included with an average follow-up period of 52.56 ± 20.4 months. Compared to the control group, patients on AP, DAPT, or VKA had significantly higher rates of minor interventions (63.1% vs. 74.4%, 79.6%, and 77.3%, respectively, p < 0.05). DAPT users exhibited a higher rate of major interventions than the control (5.6% vs. 1.3%, p = 0.053). NOAC users showed no significant difference in minor interventions compared to control and required no major interventions. Both NOAC and VKA users had significantly higher rates of recurrent epistaxis events and prolonged hospitalization compared to the control (p < 0.01 and p < 0.05, respectively).

**Conclusions:**

NOAC demonstrated more favorable outcomes than VKA in patients with acute epistaxis, and DAPT use was associated with an increased need for major interventions. These findings suggest a more conservative approach in NOAC users than other AC/AP agents.

**Supplementary Information:**

The online version contains supplementary material available at 10.1007/s00405-024-08718-6.

## Introduction

Acute epistaxis is a prevalent medical condition ranging from mild and self-limiting to severe and life-threatening [1, 2]. The etiology of epistaxis is multifactorial, with a significant proportion of cases attributed to mucosal trauma, vascular abnormalities, or systemic medical conditions. One key factor that has garnered increasing attention in recent years is the influence of anticoagulant (AC) and antiplatelet (AP) therapy on the incidence, severity, and management of acute epistaxis.

AC medications, including vitamin K antagonists (VKA, e.g., warfarin, enoxaparin), new oral anticoagulants (NOAC), and AP (e.g., aspirin, clopidogrel), are prescribed widely for the prevention and treatment of thromboembolic diseases. The primary mechanism of action for these agents involves inhibiting the clotting cascade, thereby reducing the risk of thrombus formation. However, the anticoagulant effects of these medications raise concerns regarding their potential to exacerbate epistaxis episodes.

The relationship between AC or AP and epistaxis was previously investigated [3–5]. While these medications play a vital role in preventing potentially life-threatening cardiovascular events, such as stroke and myocardial infarction, they may increase the risk of bleeding, including from the nasal vasculature. Therefore, navigating this delicate balance is crucial for optimizing patient care. Since their introduction in Israel in 2011, NOAC have become the cornerstone of AC treatment [6, 20], virtually replacing VKA. Most NOAC act through direct factor Xa inhibition, and unlike VKA, and most agents lack a readily available reversal agent, potentially leading to challenges in controlling bleeding events [7]. While several studies have investigated the relationship between NOAC and acute epistaxis [8–10], their cohorts were limited in size and follow-up duration, probably due to the relatively recent introduction of NOAC. This study evaluates the association between different AC/AP agents, particularly VKA and NOAC, and a range of acute epistaxis outcomes. Our study hypothesis was that NOAC users will demonstrate better outcomes than VKA users.

## Methods

### Study population

Records were extracted from the institute’s electronic medical records (EMR) of all patients who presented with acute epistaxis to the otolaryngology emergency department at a university-affiliated medical center (Kaplan Medical Center, Rehovot, Israel) between 2014 and 2022. The study population was divided into three groups based on the pre-existing medical treatment: AP, AC, and no AP/AC treatment (control). AP group was subdivided into aspirin, clopidogrel (Plavix^®^), and DAPT (aspirin and clopidogrel). AC group was subdivided into NOAC and VKA. The NOAC subgroup included apixaban (Eliquis^®^), rivaroxaban (Xarelto^®^), and dabigatran (Pradaxa^®^), and the VKA subgroup included warfarin (Coumadin^®^) and enoxaparin (Clexane^®^).

Patients were excluded if they met any of the following criteria: age less than 18 years, prior sinonasal surgery, hematological diseases or coagulopathies (e.g., von-Willibrand disease), autoimmune disorders (e.g., vasculitis), nasal mucosal disorders (including hereditary hemorrhagic telangiectasia—HHT), sinonasal tumors or incomplete data in their records.

### Outcome measures

The primary outcomes of this study were the interventions employed to manage acute epistaxis events. These interventions were categorized into minor (chemical or electrical cauterization, nasal tamponade) and major (endoscopic ligation under general anesthesia and embolization).

Secondary outcomes included significant bleeding events, prolonged admission, and recurrent events. *Significant bleeding* was defined as epistaxis events with any of the following: > 1 mg/dL decrease in Hb or need for blood transfusion. *Prolonged admission* was defined as admission exceeding three days (including admission and discharge days). *Recurrent events* were defined as patients returning to the emergency department with acute epistaxis during the study period.

### Data collection

The collected data included patients’ age at diagnosis, sex, past medical and surgical history, blood pressure upon admission, epistaxis characteristics including site and recurrent events, type of AC/AP medications (including dosage) and the indication for treatment, laboratory tests (complete blood count, coagulation studies), treatment modalities, need for blood transfusions, and admission rates and duration. International normalized ratio (INR) was measured in all warfarin users (normal range 2.0–3.0 and 3.5 for patients with a prosthetic heart valve).

### Statistical analyses

The association between the anti-thrombotic agent groups and various interventions or epistaxis outcomes was assessed using chi-square and Student's t-test analyses. A multivariate binary logistic regression analysis was conducted, adjusting for age, sex, and other relevant co-morbidities. Results were reported as odds ratios (OR) with 95% confidence intervals (CIs). All presented p-values are two-tailed, with significance considered at the p < 0.05 level. Statistical analyses were performed using SPSS software (IBM SPSS for Windows, Version 26.0, Armonk, NY, USA).

## Results

**Fig. 1:**
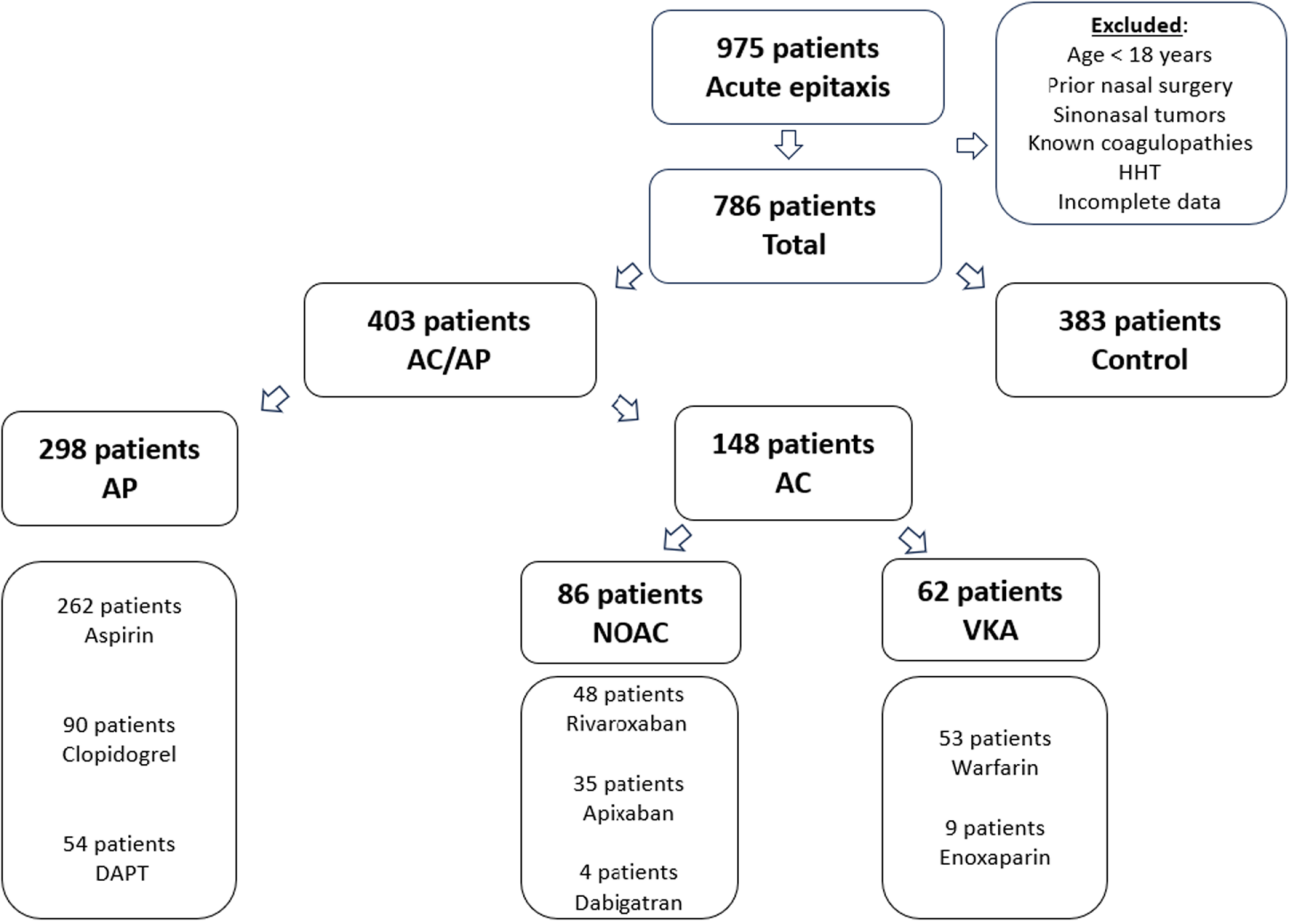
Study Flowchart ^. Shows the different study groups according to their regular anticoagulant/antiplatelet agents. ^ Overlap exists between some AP and AC users. *HHT* Hereditary Hemorrhagic Telangiectasia, *AC* anticoagulants, *AP* antiplatelets, *DAPT* Dual Antiplatelet Therapy, *NOAC* New Oral Anticoagulants, *VKA* Vitamin K antagonists

Patients’ demographics and general characteristics are presented in Table 1**.** A total of 786 patients were included, with a mean follow-up of 52.56 ± 20.4 months. AC/AP group included 403 (51.3%). Of them, 298 (37.9%) were AP users, subdivided into aspirin, clopidogrel, and DAPT, and 148 (18.8%) were AC users, subdivided into NOAC and VKA (Fig. 1). The control group included 383 (48.7%) patients. Notably, NOAC users were the oldest (77 ± 10.2 years) and had the highest rate of essential hypertension than all other groups (91.0%, p < 0.01). Among warfarin users, 14 (28.6%) patients had suprathreshold INR values. The main indication for AC treatment was atrial fibrillation (Supplemental Table 1).

**Table 1:** Patients demographics and acute epistaxis characteristics on admission

	Total	Control	Aspirin	Clopidogrel	DAPT	Warfarin	Enoxaparin	NOAC (total)	Apixaban	Rivaroxaban	Dabigatran
Patients, N (%)	786	383 (48.5)	262 (33.4)	90 (11.6)	54 (6.9)	53 (6.9)	9 (1.1)	86 (11.2)	35 (4.4)	48 (6.4)	4 (0.5)
Age (mean ± SD)	61.46 ± 20.51	49.30 ± 20.9†	72.09 ± 11.28†	73.86 ± 10.44 †	73.82 ± 10.56†	72.55 ± 9.6†	68.44 ± 20.85†	77.10 ± 10.2 †	80.44 ± 7.27 †	74.55 ± 11.51†	81.75 ± 3.6 †
Male N (%)	435 (55)	203 (52.6)	165 (62.0)*	66 (71.7) †	44 (80.0)†	36 (65.5)	4 (44.4)	39 (44.3)	14 (40)	23 (46.0)	2 (50.0)
Hypertension N (%)	461 (58.7)	118 (30.6)	224 (84.2)†	83 (90.2) †	48 (87.3)†	44 (80.0)†	5 (55.6)†	81 (91.0) †	32 (91.4) †	46 (92.2)†	4 (100.0)†
Systolic BP, admission (mean ± SD)	144.53 ± 24.55	139.7 ± 22.6	149.85 ± 23.44†	146.73 ± 22.98*	148.63 ± 24.20*	150 ± 22.0 †	137.63 ± 19.38	147.95 ± 24.38†	146.23 ± 25.85	149.6 ± 24.6†	141.67 ± 8.73
Hb (mg/dL), admission, (mean ± SD)	12.89 ± 1.94	13.3 ± 1.76	12.85 ± 2.04*	12.65 ± 2.07*	12.84 ± 2.22	12.45 ± 1.76†	11.27 ± 2.21*	12.0 ± 1.94†	12.0 ± 1.61†	11.9 ± 2.15†	13.53 ± 1.87
INR, admission, (mean ± SD)**	–	–	–	–	–	2.92 ± 1.68	–	–	–	–	–
Suprathreshold INR, N (%)	–	–	–	–	–	14 (26.4)	–	–	–	–	–
Epistaxis Location											
Anterior, N (%)	537 (68.3)	261(68.63)	185 (70.6)	60 (66.7)	36 (66.8)	34 (64.2)	7 (77.8)	57 (66.3)	21 (60.0)	33 (68.7)	3 (75.0)
Posterior, N (%)	60 (7.6)	31 (8.1)	19 (7.2)	5 (5.6)	4 (7.4)	7 (13.2)	0 (0.0)	5 (5.8)	2 (5.7)	3 (6.3)	0 (0.0)
Unspecified, N (%)	188 (23.9)	90 (23.6)	58 (22.3)	25 (27.8)	14 (29.5)	12 (22.6)	2 (22.2)	24 (27.9)	12 (34.3)	12 (25.5)	1 (25.0)
Follow-up period, months (mean ± SD)	52.56 ± 20.4	53.4 ± 18.36	57.6 ± 1.68	54.12 ± 20.16	46.2 ± 28.8†	46.92 ± 24*	61.8 ± 25.32†	42.36 ± 22.68†	34.2 ± 22.92†	46.92 ± 20.88*	53.76 ± 29.4

Demographic details and epistaxis characteristics of patients who presented to the emergency room with acute epistaxis. one patient was treated with both apixaban and rivaroxaban

*DAPT* Dual Antiplatelet Therapy, *NOAC* New Oral Anticoagulants, *INR* International Normalized Ratio

^**^INR was calculated only for warfarin users therefore cannot compare groups

^*^P < 0.05

^†^P < 0.01

**Fig. 2:**
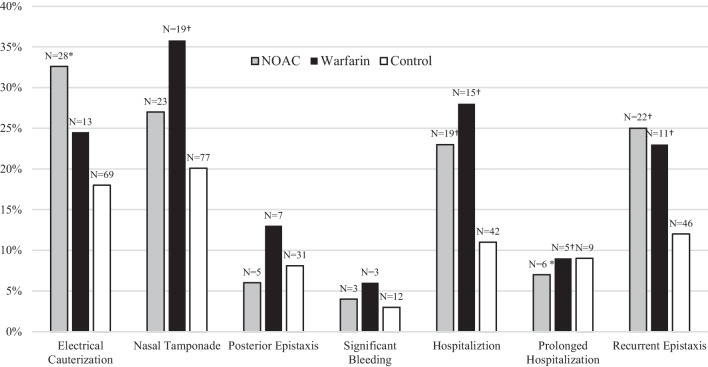
Acute Epistaxis Outcomes—NOAC and Warfarin Vs. Control. Figure 2 shows the different clinical outcomes of acute epistaxis in patients treated with NOAC, Warfarin, and without AC/AP treatment (control). *NOAC* New Oral Anticoagulants, *AC/AP* anticoagulants/antiplatelets. *P < 0.05. †P < 0.01

Epistaxis outcomes are detailed in Table 2. Patients treated with aspirin, DAPT, or warfarin demonstrated significantly higher rates of minor interventions than the control group (74.4%, 79.6%, and 77.3% vs. 63.1%, respectively. p < 0.05). Specifically, the need for nasal tamponade was significantly higher in aspirin, clopidogrel, DAPT, warfarin, and enoxaparin users compared to control (30.5%, 33.3%, 35.1%, 35.8%, 55.5% vs. 20.1%, respectively, p < 0.01). On the other hand, NOAC users did not show significant differences in minor intervention rates compared to control (70.9% vs. 63.1%, respectively, p > 0.05), including tamponade (26.6% vs 20.1%, p > 0.05). Higher rates of major interventions were noticed in DAPT users compared to the control (5.6% vs. 1.6%, p = 0.053). Compared to the control group, all AC/AP agents except enoxaparin were associated with significantly higher rates of recurrent epistaxis events. Figure 2 compares NOAC and warfarin. Although NOAC users showed trends towards lower rates of nasal tamponade, significant bleeding, admission rates, and prolonged duration of admission compared to warfarin, these differences were not statistically significant (p > 0.05). Differences regarding epistaxis outcomes between apixaban and rivaroxaban are found in Table 3. We noted a higher rate of recurrent epistaxis in the high-dose apixaban subgroup compared to the low-dose subgroup (6 [55%] vs. 3[13%], respectively, p = 0.01), and a higher rate of significant bleeding in apixaban users compared to rivaroxaban users (3 [8.8%], vs. 0 [0%], respectively, p = 0.04).

**Table 2:** Epistaxis outcomes, antiplatelet and anticoagulant agents Vs. control

	Total N = 786	Control N = 383	Aspirin N = 262	Clopidogrel N = 90	DAPT N = 54	Warfarin N = 53	Enoxaparin N = 9	NOAC (total) N = 86	Apixaban N = 35	Rivaroxaban N = 48	Dabigatran N = 4
Minor interventions (total), N (%) **^**	539 (68.6)	242 (63.1)	195 (74.4)*	65 (72.2)	43 (79.6)*	41 (77.3)*	8 (88.8)	61 (70.9)	25 (71.4)	34 (70.8)	2 (50.0)
Nasal tamponade, N (%)	202 (26.7)	77 (20.1)	80 (30.5)**†**	30 (33.3)**†**	19 (35.1)**†**	19 (35.8)**†**	5 (55.5)**†**	23 (26.7)	9 (25.7)	14 (29.1)	0 (0)
Chemical cauterization, N (%)	254 (32.3)	141 (36.8)	75 (28.6)*	21 (23.3)*	12 (22.2) *	15 (28.3)	3 (37.5)	22 (25.6)*	12 (34.2)	9 (18.7)*	1 (25)
Electrical cauterization, N (%)	184 (23.4)	69 (18.0)	72 (27.4)**†**	27 (30.0)*	20 (37.0)**†**	13 (24.5)	5 (55.6)**†**	28 (32.6)**†**	10 (28.6)	17 (35.4)**†**	1 (25.0)
Major interventions (total) N (%)	10 (1.3)	6 (1.6)	4 (1.5)	3 (3.3)	3 (5.6)	0 (0)	0 (0)	0 (0)	0 (0)	0 (0)	0 (0)
Surgery, N (%)	10 (1.3)	6 (1.6)	4 (1.5)	3 (3.3)	3 (5.6)	0 (0)	0 (0)	0 (0)	0 (0)	0 (0)	0 (0)
Embolization, N (%)	0 (0)	0 (0)	0 (0)	0 (0)	0 (0)	0 (0)	0 (0)	0 (0)	0 (0)	0 (0)	0 (0)
Blood transfusion, N (%)	5 (0.6)	3 (0.8)	1 (0.4)	0 (0)	0 (0)	0 (0)	0 (0)	1 (1.2)	0 (0)	1 (2)	0 (0)
Bleeding > 1g (Hb), N (%)	23 (3)	12 (3)	4 (1.5)	6 (7)	3 (6)	3 (6)	0 (0)	3 (4)	3 (9)	0 (0)	0 (0)
Hospitalization, N (%)	134 (17.0)	42 (11.0)	58 (22.1) **†**	23 (25.6)**†**	16 (29.6)**†**	15 (28.3)**†**	2 (22.2)	19 (22.1) **†**	8 (22.9)*	11 (22.9)*	0 (0)
Prolonged hospitalization, N (%)	38 (4.8)	9 (2.3)	18 (6.9)	12 (13.3)**†**	7 (13.0)**†**	5 (9.4)**†**	1 (11.1)	6 (7.0)*	1 (2.86)	5 (10.4)**†**	0 (0)
Recurrent visits, N (%)	136 (17.3)	46 (12.0)	56 (21.3)**†**	22 (24.4)*	12 (22.2)*	11 (20.7)**†**	2 (22.2)	22 (25.6)**†**	9 (25.7)*	12 (25.0)**†**	1 (25.0)
Time following last visit, days (mean ± SD)	126.24 ± 345.13	71 ± 366	138.91 ± 253.02	200.4 ± 346	125.84 ± 187.39	18.64 ± 20.2	162 ± 141.42	264.7 ± 515	52 ± 57.6	397.63 ± 670*	712

Compares the clinical outcomes of acute epistaxis between patients with different AC/AP treatment and patients without AC/AP treatment (control)

*DAPT* Dual Antiplatelet Therapy, *NOAC* New Oral Anticoagulants, *AC/AP* anticoagulant/antiplatelet

**^**Some patients had overlapping minor interventions

^*^P < 0.05

^†^P < 0.01

**Table 3:** Epistaxis outcomes, apixaban vs. rivaroxaban

Daily dosage		Apixaban			Rivaroxaban		
		5 mg N = 23	10 mg N = 11	Total N = 34	15 mg N = 27	20 mg N = 18	Total N = 45
Location of epistaxis, N (%)	Anterior	15 (65.2)	6 (54.5)	21 (61.8)	18 (66.7)	12 (66.7)	33 (7.3)
	Posterior	1 (4.3)	1 (9.1)	2 (5.9)	1 (3.7)	2 (1.1)	3 (6.6)
	Unknown	7 (30.0)	4 (36.4)	12 (35.3)	8 (30.0)	4 (22.2)	12 (26.6)
Minor interventions, N (%)	Electrical cauterization	5 (21.7)	5 (45.5)	10 (29.4)	10 (37.0)	5 (27.8)	17 (37.8)
	Nasal tamponade	5 (21.7)	4 (36.4)	9 (26.5)	7 (25.9)	6 (33.3)	14 (31.1)
Major interventions, N (%)	Surgery	0 (0)	0 (0)	0 (0)	0 (0)	0 (0)	0 (0)
	Embolization	0 (0)	0 (0)	0 (0)	0 (0)	0 (0)	0 (0)
Significant bleeding, N (%)		1 (4.3)	2 (18.2)	3 (8.8) *	0 (0)	0 (0)	0 (0)
Hospitalization, N (%)		4 (17.4)	4 (36.4)	8 (23.5)	6 (22.2)	4 (22.2)	11 (24.4)
Prolonged hospitalization rate, N (%)		0 (0)	1 (0)	1 (3)	2 (7)	2 (11)	5 (10)
Recurrent events, N (%)		3 (13.0)	6 (54.5)†	9 (26.5)	4 (14.8)	6 (33.3)	11 (24.4)

Compares the different clinical outcomes of acute epistaxis in patients under different NOAC agents and regimens

*NOAC* New Oral Anticoagulants

^*^P = 0.04

^†^P = 0.01

In a multivariate Cox proportional hazards regression model adjusting for age, sex, essential hypertension, diabetes, cardiovascular disease status, and chronic renal failure (Supplemental Table 2), only age remained an independent risk factor for minor intervention across all analysed AC/AP agents. The multivariate analysis revealed no significant differences in the need for intervention between AC/AP and the control group. In addition, NOAC showed significantly lower admission rates compared to the control group (HR 0.389, p < 0.05, CI [0.158–0.956]) (Table 4). This advantage was not observed for DAPT or warfarin.

**Table 4:** Association between anti-thrombotic agent with hospitalization and intervention rates as evaluated by multivariate cox proportional hazards model^a^

	Hospitalization			Minor intervention		
Agent	HR	CI (95%)	P-Value	HR	CI (95%)	P-Value
DAPT^b^	0.595	0.207–1.715	0.337	2.457	0.842–7.171	0.100
Warfarin^b^	0.418	0.167–1.049	0.063	1.758	0.705–4.381	0.226
NOAC^b^	**0.389**	**0.158–0.956**	**0.04**	1.969	0.846–4.583	0.116

Bold: Significance rates were considered when P < 0.05

Describes the association between anti-thrombotic agent and hospitalization and minor intervention rates using a multivariate cox proportional hazard model

*HR* Hazard Ratio, *CI* Confidence Interval, *DAPT* Dual Antiplatelet Therapy, *VKA* Vitamin K antagonists, *NOAC* New Oral Anticoagulants

^a^The model included the following variables: age, sex, diabetes, chronic renal failure, hypertension, and ischemic heart disease

^b^Versus the control group

## Discussion

Acute epistaxis is one of the leading causes of visits to the otolaryngology emergency room. While it is well known that AC/AP treatment increases the risk for epistaxis [9–11], this study aimed to describe the specific impact of different AC/AP agents on acute epistaxis outcomes. Our results suggest a favorable outcome profile for NOAC compared to VKA in acute epistaxis. Additionally, we identified an elevated risk of severe epistaxis requiring major interventions associated with DAPT.

### Demographics and epistaxis characteristics

Patients receiving AC/AP treatment constitute 51.2% of our study population, aligning with findings from prior research [11, 12]. Notably, NOAC users, particularly those in the apixaban subgroup, were the oldest and exhibited elevated rates of essential hypertension, potentially heightening the susceptibility to epistaxis. The difficulty in reaching appropriate INR values highlights one of the disadvantages of warfarin compared to NOAC in acute epistaxis. A significant drawback associated with warfarin use is the continual necessity for INR monitoring. Within our cohort, 14 (26.4%) patients displayed supra-threshold INR values, augmenting the risk of persistent epistaxis. Furthermore, we observed that 20 (37.7%) patients had low INR values upon admission, possibly attributable to the independent discontinuation of warfarin due to recurring epistaxis episodes. In fact, the majority (64.1%) of warfarin users exhibited inappropriate INR values, surpassing the rates in previous studies [13]. The challenge in achieving suitable INR values underscores a notable drawback of warfarin compared to NOAC in acute epistaxis.

### Recurrent epistaxis

All AC/AP users except for enoxaparin demonstrated significantly higher recurrence rates than the control, which aligns with previous studies [9, 11, 14]. Interestingly, Glikson et al. reported lower recurrence in NOAC than warfarin [10], but this study had a relatively short follow-up period. In our cohort, we showed that patients treated with rivaroxaban had significantly longer intervals between ER admissions due to acute epistaxis compared to control.

### Interventions and significant bleeding rates

As most cases were classified as anterior epistaxis (68.3%), minor interventions were usually sufficient to control this bleeding. Patients with aspirin, DAPT, and warfarin demonstrated significantly higher rates of minor interventions necessary for epistaxis control compared to the control group. Importantly, patients with NOAC did not have different rates of minor interventions, including nasal tamponade, compared to the general population. Except for being highly inconvenient to the patient and increasing the risk of mucosal trauma and subsequent synechia and hemorrhage, nasal tamponade may also lead to prolonged hospitalization, as the standard practice in our institution is to wait 48 h until tampon extraction. Therefore, nasal tamponade is reserved for persistent epistaxis that does not resolve after more subtle measures such as local pressure or cauterization and in cases of posterior source or diffuse bleeding. Accordingly, our data show that chemical cauterization was more prevalent in the control group, unlike electrical cauterization, which was more prevalent in the AP and NOAC groups, reflecting more persistent bleeding in the latter groups compared to the control.

Interestingly, no patient in the VKA or NOAC subgroups required major intervention for epistaxis control. Near-significant higher rates of major interventions were noted in the DAPT group (p = 0.053). When examining the parameters of ‘significant bleeding,’ our data show no differences between the groups, unlike previous studies showing higher rates of significant bleeding VKA compared to NOAC [9]. The relatively low rates of significant bleeding and major interventions can be explained by two main reasons. First, our department’s conservative approach prefers nasal tamponade or in-office procedures such as cauterization under local anesthesia (even in cases of posterior epistaxis) rather than ligation under general anesthesia. Second, our hospital is a referral center for large community healthcare services; therefore, most acute epistaxis cases we encounter are usually minor and rarely necessitate surgery or embolization.

### Hospitalization rates and length

Hospitalization rates were significantly higher in all patients with AC/AP treatment, without prominent differences between VKA and NOAC. However, we did observe a shorter length of hospitalization in NOAC, which may be explained by the fact that fewer patients with NOAC needed nasal tamponade, as previously discussed. Similar results are found in previous studies comparing NOAC to VKA [11, 14, 15]. Sauter et al. also described lower hospitalization rates in patients treated with rivaroxaban [16], attributed to its shorter half-life and easier control of bleeding episodes. These findings correspond well with our multivariate analysis results showing significantly lower hospitalization rates among NOAC patients than control.

### Warfarin Vs. NOAC

In their vast population-based study, Ingason et al. investigated epistaxis rates in patients treated with warfarin and NOAC in Iceland [17]. A total of 7,081 patients with AC treatment were followed for five years, and epistaxis events were documented for analysis. Their results showed that the risk for epistaxis was more than twofold in patients under warfarin compared to apixaban and rivaroxaban (2.2 events per 100 patient years vs. 0.6 and 1.0, respectively, p < 0.001). Due to the design of our study, we could not calculate the relative risk for epistaxis in different AC agents. However, we did show that warfarin was associated with higher rates of interventions and prolonged hospitalization compared to NOAC.

Despite their comprehensive initial cohort, the Icelandic study included only 93 cases of acute epistaxis with a ~ 10% admission rate (11 patients) which is lower than our cohort and, represents a relatively low absolute number of hospitalized patients.

In our study, we noticed important differences in patients’ characteristics; compared to warfarin, patients with NOAC were older and with higher percentage of hypertension, increasing their frailty and risk for epistaxis [18]. However, epistaxis events in NOAC users were easier to treat and generally led to better outcomes than warfarin.

### Apixaban Vs. rivaroxaban

A previous study found that rivaroxaban was associated with a higher risk for epistaxis than apixaban [17], as well as a higher rate of significant bleeding and gastrointestinal bleeding [19]. On the contrary, our data show a higher rate of significant bleeding in the apixaban group. In addition, we did not find any dose-dependent differences in either apixaban or rivaroxaban, except for higher hospitalization rates in the high-dose apixaban group (10 mg daily) compared to the low-dose group (5 mg daily). This is in concordance with previous studies [17].

### Limitations

This retrospective study acknowledges inherent limitations that may introduce information bias. To address this, thorough double verification of patient records was conducted. Additionally, our analysis lacked information regarding AC/AP discontinuation during hospitalization. In our practice, withholding AC/AP is individualized and not guided by a strict uniform protocol. Typically, discontinuation is considered for significant bleeding or recurrent events only after consultation with a cardiologist.

## Conclusions

Our findings suggest a potential benefit of NOAC compared to other AC/AP agents regarding the severity of acute epistaxis. NOAC demonstrated favorable outcomes over VKA, whereas DAPT use was associated with a higher risk of severe bleeding requiring major interventions.

No major differences were found between different NOAC agents or dosing regimens. These results suggest that a more conservative approach may be warranted for NOAC users, thereby avoiding unnecessary interventions and hospitalizations.

## Supplementary Information

Additional file1 (DOCX 17 KB)

## Data Availability

The data supporting this study's findings are not openly available due to sensitivity reasons and are available from the corresponding author upon reasonable request. Data are located in controlled access data storage at Kaplan Medical Center.
