# Supplementary material for: The impact of different anticoagulants and antiplatelets regimens on acute epistaxis outcomes
Source: Eur Arch Otorhinolaryngol. 2024 May 23;281(9):4863–71. doi: 10.1007/s00405-024-08718-6 (PMC11393048; doi:10.1007/s00405-024-08718-6)
Supplement: Supplementary file 1 — Additional file1 (DOCX 17 KB) [file 405_2024_8718_MOESM1_ESM.docx]

**Supplemental Table 1 – Main Indication for Anticoagulant Therapy**

|  | **Warfarin** | **NOAC** |
| --- | --- | --- |
| Atrial fibrillation, N (%) | 26 (49.0) | 73 (85.0) |
| TIA/CVA, N (%) | 7 (13.2) | 8 (9.3) |
| Prosthetic Heart Valve, N (%) | 12 (22.6) | 1 (1.2) |
| PE/DVT, N (%) | 4 (7.5) | 3 (3.5) |
| Unknown, N (%) | 4 (7.5) | 1 (1.2) |
| Total, N (%) | 53 (100.0) | 86 (100.0) |

**NOAC** = New Oral Anticoagulants, **TIA/CVA** = Transient Ischemic Attack, Cerebrovascular Accident, **PE/DVT** = Pulmonary Embolism, Deep vein Thrombosis.

|  | Hospitalization | | | | | | Minor intervention | | | | | |
| --- | --- | --- | --- | --- | --- | --- | --- | --- | --- | --- | --- | --- |
|  | DAPT | | Warfarin | | NOAC | | DAPT | | Warfarin | | NOACS | |
|  | HR  (95% CI) | P-value | HR  (95% CI) | P-value | HR  (95% CI) | P-value | HR  (95% CI) | P-value | HR  (95% CI) | P-value | HR  (95% CI) | P-value |
| Age | 0.98  (0.96-1.0) | 0.06 | 0.98  (0.97-1.0) | 0.09 | 0.98  (0.96-0.99) | **0.01** | 1.017  (1.0-1.03) | **0.01** | 1.02  (1.00-1.04) | **0.002** | 1.017  (1.0-1.03) | **0.01** |
| Gender (male) | 0.77  (0.41- 1.43) | 0.40 | 0.98  (0.55-1.75) | 0.94 | 0.67  (0.38-1.19) | 0.17 | 1.08  (0.71-1.64) | 0.72 | 1.15  (0.77-1.73) | 0.49 | 1.19  (0.80-1.77) | 0.39 |
| Hypertension | 0.85  (0.39-1.85) | 0.68 | 1.10  (0.51-2.39) | 0.80 | 0.89  (0.42-1.91) | 0.77 | 1.40  (0.77-2.52) | 0.27 | 1.62  (0.9-2.94) | 0.10 | 1.21  (0.68-2.16) | 0.52 |
| Diabetes | 0.95  (0.43- 2.12) | 0.90 | 1.09  (0.48-2.42) | 0.85 | 1.10  (0.52-2.31) | 0.80 | 1.18  (0.60-2.34) | 0.64 | 1.06  (0.53-2.11) | 0.87 | 1.21  (0.66-2.24) | 0.54 |
| IHD | 1.81  (0.60-5.44) | 0.29 | 0.89  (0.32- 2.51) | 0.83 | 0.48  (0.18- 1.29) | 0.14 | 1.23  (0.42-3.59) | 0.70 | 1.41  (0.53-3.73) | 0.49 | 2.27  (0.95-5.41) | 0.07 |
| CRF | 1.06  (0.29- 3.86) | 0.93 | 0.78  (0.23-2.62) | 0.68 | 0.30  (0.06- 1.37) | 0.12 | 0.85  (0.25-2.88) | 0.79 | 0.75  (0.25-2.28) | 0.61 | 1.06  (0.41-2.70) | 0.91 |
| Anti-thrombotic agent | 0.6  (0.21- 1.72) | 0.34 | 0.42  (0.171-0.50) | 0.06 | 0.39  (0.16- 0.96) | **0.04** | 2.46  (0.84-7.17) | 0.10 | 1.76  (0.70-4.38) | 0.23 | 1.97  (0.85-4.583) | 0.12 |

**Supplemental Table 2 - Multivariate Cox Proportional Hazards Regression Model**

**IHD**=Ischemic heart disease, **CRF**=Chronic Renal Failure, **DAPT**= Dual Antiplatelet Therapy, **NOAC**= New Oral Anticoagulants
